# Supplementary material for: A Critical View of the Application of the APEX Software (Aqueous Photochemistry of Environmentally-Occurring Xenobiotics) to Predict Photoreaction Kinetics in Surface Freshwaters
Source: Molecules. 2019 Dec 18;25(1):9. doi: 10.3390/molecules25010009 (PMC7017383; doi:10.3390/molecules25010009)
Supplement: Supplementary file 1 [file molecules-25-00009-s001.zip › 3.2.4_gcc-4.4.0.zip!/3.2.4_gcc-4.4.0/3.2.4_gcc-4.4.0/license/jogl/Userguide.html]

Jogl - User's Guide


# Jogl - User's Guide

- Overview- Developing with JOGL
    - Building the source tree- Local installation for development- Java Web Start integration- Applet support
    - GLDrawable and GLContext- Creating a GLAutoDrawable- Writing a GLEventListener- Using the Composable Pipeline- Heavyweight and Lightweight Issues- Multithreading Issues- Pbuffers- GLU- More Resources- Platform notes
                        - All Platforms- Windows- Linux- Solaris, Linux (X11 platforms)- Macintosh OS X
                        - Version History

## Overview

Jogl is a Java programming language binding for the OpenGL 3D graphics
API. It supports integration with the Java platform's AWT and Swing
widget sets while providing a minimal and easy-to-use API that handles
many of the issues associated with building multithreaded OpenGL
applications. Jogl provides access to the latest OpenGL routines
(OpenGL 2.0 with vendor extensions) as well as platform-independent
access to hardware-accelerated offscreen rendering ("pbuffers"). Jogl
also provides some of the most popular features introduced by other
Java bindings for OpenGL like GL4Java, LWJGL and Magician, including a
composable pipeline model which can provide faster debugging for
Java-based OpenGL applications than the analogous C program.

Jogl was designed for the most recent versions of the Java platform
and for this reason supports only J2SE 1.4 and later. It also only
supports truecolor (15 bits per pixel and higher) rendering; it does
not support color-indexed modes. It was designed with New I/O (NIO) in
mind and uses NIO internally in the implementation. The Jogl binding
is itself written almost completely in the Java programming language.
There are roughly 150 lines of handwritten C code in the entire Jogl
source base (100 of which work around bugs in older OpenGL drivers on
Windows); the rest of the native code is autogenerated during the
build process by a new tool called GlueGen, the source code of
which is available from its own java.net project.

The JOGL source tree in its current form is an experimental workspace
for the JSR-231 Java
Bindings for OpenGL JSR. JOGL is not the official reference
implementation, but an evolving workspace. Snapshots of the JOGL
source tree are run through the JSR-231 Technology Compatibility Kit
(TCK) to become the official reference implementation (RI). As of this
writing the JSR has not been finalized, so the first official RI of
the JSR has not yet been produced.

## Developing with JOGL

### Building the source tree

Most developers using JOGL will download the most current release
build. Separate instructions are available on how to build
the source tree.

### Local installation for development

The JOGL distribution for developers comes in the form of a zip
archive which contains the Java classes to call OpenGL from Java, as
well as the associated JNI native libraries. JOGL depends on some
run-time support classes and native code provided by the GlueGen
project; these classes and native code are also provided in the zip
bundles.

If you are developing a new application which uses JOGL, download the
zip archive for your platform (for example.,
jogl-[version]-windows-i586.zip) and unzip it. Modify your CLASSPATH
environment variable to include the full paths to jogl.jar and
gluegen-rt.jar; for example,
".;C:\Some\Other\Package\foo.jar;C:\Users\myhome\jogl-[version]-windows-i586\lib\jogl.jar;C:\Users\myhome\jogl-[version]-windows-i586\lib\gluegen-rt.jar".
(If you did not previously set the CLASSPATH environment variable, you
may want to make sure that ".", the current directory, is on your new
CLASSPATH.) Modify your PATH environment variable (Windows),
LD\_LIBRARY\_PATH environment variable (Solaris and Linux), or
DYLD\_LIBRARY\_PATH environment variable (Mac OS X) to contain the full
path to the "lib" directory; for example, on Windows, add
"C:\Users\myhome\jogl-[version]-windows-i586\lib" to your PATH using
the System control panel, Advanced tab, Environment Variables
button. At this point your Java installation should be able to see the
JOGL class files. Users of IDEs such as NetBeans and Eclipse should
consult the IDE's documentation to see how to add jar files and native
libraries to their current project.

Dropping the JOGL jar and native library into the extension directory
of the JRE is **strongly discouraged**. Doing so can cause
conflicts with third-party applications launched via Java Web Start,
and causes confusion later when upgrading the distribution.

If you are on the Linux platform, please see the Linux-specific
platform notes, below, with information on incompatibility between the
JPackage Java RPMs and JOGL.

### Java Web Start integration

The recommended distribution vehicle for applications using JOGL is
Java Web Start. JOGL-based applications do not even need to be signed;
all that is necessary is to reference the JOGL extension JNLP file.
Because the JOGL jar files are signed, an unsigned application can
reference the signed JOGL library and continue to run inside the
sandbox.

To reference JOGL within your application's JNLP file, simply place
the following line in the `<resources>` section:

```
  <extension name="jogl" href="http://download.java.net/media/jogl/builds/archive/jsr-231-webstart-current/jogl.jnlp" />
```

This JNLP file points to the current JSR-231 unofficial development
build. For reference, the extension JNLP file for the most recent
official JSR-231 build is available at

```
  <extension name="jogl" href="http://download.java.net/media/jogl/builds/archive/jsr-231-1.1.0/webstart/jogl.jnlp" />
```

Note that before JOGL transitioned to the JSR-231 APIs, there were
releases of the library in the `net.java.games.jogl`
namespace under version numbers "1.0", "1.1", and "1.1.1". All of
these releases have been superseded by JSR-231. Please update your
applications.

### Applet support

Lilian Chamontin, in conjunction with several other members of the
JOGL community, has contributed a JOGL applet installer. This
installer uses some clever tricks to allow deployment of unsigned
applets which use JOGL into existing web browsers and JREs as far back
as 1.4.2, which is the earliest version of Java supported by JOGL.

The JOGLAppletInstaller is distributed inside jogl.jar as a utility
class in com.sun.opengl.util. It requires that the developer host a
local, signed copy of jogl.jar and all of the jogl-natives jars; the
certificates must be the same on all of these jars. Note that in the
release builds of JOGL all of these jars are signed by Sun
Microsystems, so the developer can deploy applets without needing any
certificates.

The JOGLAppletInstaller javadoc describes the basic steps for
deployment of an applet utilizing JOGL. Please refer to this
documentation for more information. A live example of deploying an
unsigned JOGL applet will be added to this documentation shortly once
the first signed build of the JOGLAppletInstaller has been shipped.

## GLDrawable and GLContext

The JSR-231 APIs specify interfaces two low-level OpenGL abstractions:
drawables and contexts. An OpenGL drawable is effectively a surface
upon which OpenGL rendering will be performed. In order to perform
rendering, an OpenGL rendering context is needed. Contexts and
drawables typically go hand-in-hand. More than one context may be
created for a particular drawable. In the JSR-231 abstractions, a
context is always associated with exactly one drawable.

Most end users will not need to use these abstractions directly.
However, when sharing textures, display lists and other OpenGL objects
between widgets, the concrete identifier for the "namespace" for these
objects is the GLContext.

## Creating a GLAutoDrawable

Jogl provides two basic widgets into which OpenGL rendering can be
performed. The GLCanvas is a heavyweight AWT widget which supports
hardware acceleration and which is intended to be the primary widget
used by applications. The GLJPanel is a fully Swing-compatible
lightweight widget which supports hardware acceleration but which is
not as fast as the GLCanvas because it typically reads back the frame
buffer in order to draw it using Java2D. The GLJPanel is intended to
provide 100% correct Swing integration in the circumstances where a
GLCanvas can not be used. See this
article on The
Swing Connection for more information about mixing lightweight and
heavyweight widgets. See also the section on "Heavyweight and
Lightweight Issues" below. Recent work in the Mustang release of the
JDK has sped up the GLJPanel significantly when the Java2D OpenGL
pipeline is enabled; see this
forum discussion for more details.

Both the GLCanvas and GLJPanel implement a common interface called
GLAutoDrawable so applications can switch between them with minimal
code changes. The GLAutoDrawable interface provides

- access to the GL object for calling OpenGL routines- a callback mechanism (GLEventListener) for performing OpenGL
    rendering- a `display()` method for forcing OpenGL rendering to
      be performed synchronously- AWT- and Swing-independent abstractions for getting and setting
        the size of the widget and adding and removing event listeners

When creating GLCanvas and GLJPanel instances, the user may request a
certain set of OpenGL parameters in the form of a GLCapabilities
object, customize the format selection algorithm by specifying a
GLCapabilitiesChooser, share textures and display lists with other
GLDrawables, and specify the display device on which the
GLAutoDrawable will be created (GLCanvas only).

A GLCapabilities object specifies the OpenGL parameters for a
newly-created widget, such as the color, alpha,, z-buffer and
accumulation buffer bit depths and whether the widget is
double-buffered. The default capabilities are loosely specified but
provide for truecolor RGB, a reasonably large depth buffer,
double-buffered, with no alpha, stencil, or accumulation buffers.

An application can override the default pixel format selection
algorithm by providing a GLCapabilitiesChooser to the GLCanvas or
GLJPanel constructor. (Not all platforms support the
GLCapabilitiesChooser mechanism, however; it may be ignored, in
particular on Mac OS X where pixel format selection is very different
than on other platforms.) The chooseCapabilities method will be called
with all of the available pixel formats as an array of GLCapabilities
objects, as well as the index indicating the window system's
recommended choice; it should return an integer index into this
array. The DefaultGLCapabilitiesChooser uses the window system's
recommendation when it is available, and otherwise attempts to use a
platform-independent selection algorithm.

The GLJPanel can be made non-opaque according to Swing's rendering
model, so it can act as an overlay to other Swing or Java2D drawing.
In order to enable this, set up your GLCapabilities object with a
non-zero alpha depth (a common value is 8 bits) and call
setOpaque(false) on the GLJPanel once it has been created. Java2D
rendering underneath it will then show through areas where OpenGL has
produced an alpha value less than 1.0. See the JGears and JRefract
demos for examples of how to use this functionality.

## Writing a GLEventListener

Applications implement the GLEventListener interface to perform OpenGL
drawing via callbacks. When the methods of the GLEventListener are
called, the underlying OpenGL context associated with the drawable is
already current. The listener fetches the GL object out of the
GLAutoDrawable and begins to perform rendering.

The `init()` method is called when a new OpenGL context is
created for the given GLAutoDrawable. Any display lists or textures
used during the application's normal rendering loop can be safely
initialized in `init()`. It is important to note that
because the underlying AWT window may be destroyed and recreated while
using the same GLCanvas and GLEventListener, the GLEventListener's
`init()` method may be called more than once during the
lifetime of the application. The init() method should therefore be
kept as short as possible and only contain the OpenGL initialization
required for the `display()` method to run properly. It is
the responsibility of the application to keep track of how its various
OpenGL contexts share display lists, textures and other OpenGL objects
so they can be either be reinitialized or so that reinitialization can
be skipped when the `init()` callback is called.

Note also that the GLEventListener should be added to the
GLAutoDrawable before the GLAutoDrawable is shown or rendered to for
the first time. If this is not done, it is possible that the init()
method will not be called on the GLEventListener. JOGL does not
maintain internal state to keep track of whether init() has been
called on a particular GLEventListener since the last time an OpenGL
context was created for that GLAutoDrawable.

The `display()` method is called to perform per-frame
rendering. The `reshape()` method is called when the
drawable has been resized; the default implementation automatically
resizes the OpenGL viewport so often it is not necessary to do any
work in this method. The `displayChanged()` method is
designed to allow applications to support on-the-fly screen mode
switching, but support for this is not yet implemented so the body of
this method should remain empty.

It is strongly recommended that applications always refetch the GL
object out of the GLAutoDrawable upon each call to the
`init()`, `display()` and `reshape()`
methods and pass the GL object down on the stack to any drawing
routines, as opposed to storing the GL in a field and referencing it
from there. The reason is that multithreading issues inherent to the
AWT toolkit make it difficult to reason about which threads certain
operations are occurring on, and if the GL object is stored in a field
it is unfortunately too easy to accidentally make OpenGL calls from a
thread that does not have a current context. This will usually cause
the application to crash. For more information please see the section
on multithreading.

## Using the Composable Pipeline

Jogl supports the "composable pipeline" paradigm introduced by the
Magician Java binding for OpenGL. The DebugGL pipeline calls
`glGetError` after each OpenGL call, reporting any errors
found. It can greatly speed up development time because of its
fine-grained error checking as opposed to the manual error checking
usually required in OpenGL programs written in C. The TraceGL prints
logging information upon each OpenGL call and is helpful when an
application crash makes it difficult to see where the error occurred.

To use these pipelines, call `GLAutoDrawable.setGL` at the
beginning of the `init` method in your GLEventListener. For
example,

```
class MyListener implements GLEventListener {
  public void init(GLDrawable drawable) {
    drawable.setGL(new DebugGL(drawable.getGL()));
    // ...
  }

  // ...
}
```

Note that the GLAutoDrawable.setGL() method simply calls setGL() on
the default OpenGL context created by the GLAutoDrawable, so
sophisticated applications creating their own OpenGL contexts can use
the composable pipeline with these contexts by setting the GL object
in the context object itself. The composable pipeline needs to be
re-installed every time GLContext.makeCurrent() returns
CONTEXT\_CURRENT\_NEW.

## Heavyweight and Lightweight Issues

As mentioned above, JOGL supplies both a heavyweight (GLCanvas) and a
lightweight (GLJPanel) widget to be able to provide the fastest
possible performance for applications which need it as well as 100%
correct Swing integration, again for applications which need it. The
GLCanvas usually provides higher performance than the GLJPanel, though
in recent releases the GLJPanel's speed has been improved when the
Java2D/OpenGL pipeline is active as described in this
forum discussion. Nonetheless, the GLCanvas can be used in almost
every kind of application except those using JInternalFrames. Please
see the Swing Connection article mentioned above for details on mixing
heavyweight and lightweight widgets. A couple of common pitfalls are
described here.

When using JPopupMenus or Swing tool tips in conjunction with the
GLCanvas, it is necessary to disable the use of lightweight widgets
for the popups. See the methods
`ToolTipManager.setLightWeightPopupEnabled`,
`JPopupMenu.setLightWeightPopupEnabled`, and
`JPopupMenu.setDefaultLightWeightPopupEnabled`.

There are occasionally problems with certain LayoutManagers and
component configurations where if a GLCanvas is placed in the middle
of a set of lightweight widgets then it may only grow and never
shrink. These issues are documented somewhat in JOGL Issue
135 and most recently in the thread "Resize
behaviour" in the JOGL forum. The root cause is behavior of the
Canvas, and in particular its ComponentPeer. The implementation of
getPreferredSize() calls getMinimumSize() and getMinimumSize() turns
around and calls Component.getSize(). This effectively means that the
Canvas will report its preferred size as being as large as the
component has ever been. For some layout managers this doesn't seem to
matter, but for others like the BoxLayout it does. See the test case
attached to Issue 135 for an example. Replacing the GLCanvas with an
ordinary Canvas yields the same behavior.

One suggestion was to override getPreferredSize() so that if a
preferred size has not been set by the user, to default to (0,
0). This works fine for some test cases but breaks all of the other
JOGL demos because they use a different LayoutManager. There appear to
be a lot of interactions between heavyweight vs. lightweight widgets
and layout managers. One experiment which was done was to override
setSize() in GLCanvas to update the preferred size. This works down
to the size specified by the user; if the window is resized any
smeller the same problem appears. If reshape() (the base routine of
setSize(), setBounds(), etc.) is changed to do the same thing, the
demo breaks in the same way it originally did. Therefore this solution
is fragile because it isn't clear which of these methods are used
internally by the AWT and for what purposes.

There are two possible solutions, both application-specific. The best
and most portable appears to be to put the GLCanvas into a JPanel and
set the JPanel's preferred size to (0, 0). The JPanel will cause this
constraint to be enforced on its contained GLCanvas. The other
workaround is to call `setPreferredSize(new Dimension(0,
0))` on a newly-created GLCanvas; this method is new in 1.5.

Another issue that occasionally arises on Windows is flickering during
live resizing of a GLCanvas. This is caused by the AWT's repainting
the background of the Canvas and can not be overridden on a per-Canvas
basis, for example when subclassing Canvas into GLCanvas. The
repainting of the background of Canvases on Windows can be disabled by
specifying the system property
`-Dsun.awt.noerasebackground=true`. Whether to specify this
flag depends on the application and should not be done universally,
but instead on a case-by-case basis. Some more detail is in the thread
"TIP:
JOGL + Swing flicker" in the JOGL forum.

## Multithreading Issues

Jogl was designed to interoperate with the AWT, an inherently
multithreaded GUI toolkit. OpenGL, in contrast, was originally
designed in single-threaded C programming environments. For this
reason Jogl provides a framework in which it is possible to write
correct multithreaded OpenGL applications using the GLEventListener
paradigm.

If an application written using Jogl interacts in any way with the
mouse or keyboard, the AWT is processing these events and the
multithreaded aspects of the program must be considered.

OpenGL applications usually behave in one of two ways: either they
repaint only on demand, for example when mouse input comes in, or they
repaint continually, regardless of whether user input is coming in. In
the repaint-on-demand model, the application can merely call
`GLAutoDrawable.display()` manually at the end of the mouse
or keyboard listener to cause repainting to be done. Alternatively if
the application knows the concrete type of the GLDrawable it can call
repaint() to have the painting scheduled for a later time.

In the continuous repaint model, the application typically has a main
loop which is calling `GLAutoDrawable.display()`
repeatedly, or is using the Animator class, which does this
internally. In both of these cases the OpenGL rendering will be done
on this thread rather than the internal AWT event queue thread which
dispatches mouse and keyboard events.

Both of these models (repaint-on-demand and repaint continually) still
require the user to think about which thread keyboard and mouse events
are coming in on, and which thread is performing the OpenGL rendering.
OpenGL rendering **may not** occur directly inside the mouse or
keyboard handlers, because the OpenGL context for the drawable is not
current at this point (hence the warning about storing a GL object in
a field, where it can be fetched and accidentally used by another
thread). However, a mouse or keyboard listener may invoke
`GLAutoDrawable.display()`.

It is generally recommended that applications perform as little work
as possible inside their mouse and keyboard handlers to keep the GUI
responsive. However, since OpenGL commands can not be run from
directly within the mouse or keyboard event listener, the best
practice is to store off state when the listener is entered and
retrieve this state during the next call to
`GLEventListener.display()`.

Furthermore, it is recommended that if there are long computational
sequences in the GLEventListener's `display` method which
reference variables which may be being simultaneously modified by the
AWT thread (mouse and keyboard listeners) that copies of these
variables be made upon entry to `display` and these copies
be referenced throughout display() and the methods it calls. This will
prevent the values from changing while the OpenGL rendering is being
performed. Errors of this kind show up in many ways, including certain
kinds of flickering of the rendered image as certain pieces of objects
are rendered in one place and other pieces are rendered elsewhere in
the scene. Restructuring the display() method as described has solved
all instances of this kind of error that have been seen with Jogl to
date.

Prior to Jogl 1.1 b10, the Jogl library attempted to give applications
strict control over which thread or threads performed OpenGL
rendering. The `setRenderingThread()`,
`setNoAutoRedrawMode()` and `display()` APIs
were originally designed to allow the application to create its own
animation thread and avoid OpenGL context switching on platforms that
supported it. Unfortunately, serious stability issues caused by
multithreading bugs in either vendors' OpenGL drivers or in the Java
platform implementation have arisen on three of Jogl's major supported
platforms: Windows, Linux and Mac OS X. In order to address these
bugs, the threading model in Jogl 1.1 b10 and later has changed.

All GLEventListener callbacks and other internal OpenGL context
management are now performed on one thread. (In the current
implementation, this thread is the AWT event queue thread, which is a
thread internal to the implementation of the AWT and which is always
present when the AWT is being used. Future versions of Jogl may change
the thread on which the OpenGL work is performed.) When the
`GLAutoDrawable.display()` method is called from user code,
it now performs the work synchronously on the AWT event queue thread,
even if the calling thread is a different thread. The
`setRenderingThread()` optimization is now a no-op. The
`setNoAutoRedrawMode()` API still works as previously
advertised, though now that all work is done on the AWT event queue
thread it no longer needs to be used in most cases. (It was previously
useful for working around certain kinds of OpenGL driver bugs.)

Most applications will not see a change in behavior from this change
in the Jogl implementation. Applications which use thread-local
storage or complex multithreading and synchronization may see a change
in their control flow requiring code changes. While it is strongly
recommended to change such applications to work under the new
threading model, the old threading model can be used by specifying the
system property `-Djogl.1thread=auto` or
`-Djogl.1thread=false`. The "auto" setting is equivalent to
the behavior in 1.1 b09 and before, where on ATI cards the
single-threaded mode would be used. The "false' setting is equivalent
to disabling the single-threaded mode. "true" is now the default
setting.

In the JSR-231 APIs the single-threaded behavior continues to be the
default and the `setRenderingThread()` and
`setNoAutoRedrawMode()` APIs have been removed. The public
`Threading` class still provides some control over the
internal use of threads in the library as well as external access to
these mechanisms.

## Pbuffers

Jogl exposes hardware-accelerated offscreen rendering (pbuffers) with
a minimal and platform-agnostic API. Several recent demos have been
successfully ported from C/C++ to Java using Jogl's pbuffer APIs.
However, the pbuffer support in Jogl remains one of the more
experimental aspects of the package and the APIs may need to change in
the future.

To create a pbuffer, call
`GLDrawableFactory.createGLPbuffer()`. It is wise to call
`GLDrawableFactory.canCreateGLPbuffer()` first to ensure
the graphics card has pbuffer support first. The pbuffer is created
immediately and is available for rendering as soon as
`createGLPbuffer` returns.

A pbuffer is used in conjunction with the GLEventListener mechanism by
calling its display() method. Rendering, as always, occurs while the
pbuffer's OpenGL context is current. There are render-to-texture
options that can be specified in the GLCapabilities for the pbuffer
which can make it easier to operate upon the resulting pixels. These
APIs are however highly experimental and not yet implemented on all
platforms.

## GLU

Jogl contains support for the GLU (OpenGL Utility Library) version
1.3. Jogl originally supported GLU by wrapping the C version of the
APIs, but over time, and thanks to the contributions of several
individuals, it now uses a pure-Java version of SGI's GLU library. The
pure Java port is enabled by default, and addresses stability issues
on certain Linux distributions as well as the lack of native GLU 1.3
support on the Windows platform. In case of problems with the Java
port, the C version of the GLU library may be used by specifying the
system property `-Djogl.glu.nojava` on the command
line. All of the same functionality is exposed with both the Java and
C versions of the GLU library; currently NURBS support is the only
missing feature on both sides. If you run into problems with the Java
port of the GLU library please file a bug using the Issue Tracker on
the Jogl home page.

To use the GLU, simply instantiate a GLU object via `new
GLU()` at the beginning of your program. The methods on the GLU
object may be called at any point when an OpenGL context is current.
Because the GLU implementation is not thread-safe, one GLU object
should be created for each GLEventListener or other entity performing
OpenGL rendering in a given thread.

## More Resources

The JOGL
forum on javagaming.org is
the best place to ask questions about the library. Many users, as well
as the Jogl developers, read this forum frequently, and the archived
threads contain a lot of useful information (which still needs to be
distilled into documentation).

The JOGL demos provide
several examples of usage of the library.

Pepijn Van Eeckhoudt, Kevin Duling and Abdul Bezrati have done JOGL ports of
many of the the NeHe demos. These are small examples of various
pieces of OpenGL functionality. See also the NeHe web site.

Pepijn also did a JOGL port of
Paolo Martella's GLExcess
demo. To see the news update about this port, go to the main GLExcess
site and scroll down.

Gregory Pierce's introduction
to JOGL is a useful tutorial on starting to use the JOGL library.

For release information about the JOGL library, please see the JOGL Release
Information thread on the JOGL forum on javagaming.org.

Please post on the JOGL forum if you have a resource you'd like to add
to this documentation.

## Platform Notes

### All Platforms

The following issues, among others, are outstanding on all platforms:

- A few remaining stability issues, mostly on older graphics cards.- JOGL now supports experimental integration and interoperability
    with the Java2D/OpenGL pipeline in Java SE 6 (Mustang), enabling a
    much faster GLJPanel as well as other features. Please see this
    forum discussion for more details.

### Windows

For correct operation, it is necessary to specify the system property
`-Dsun.java2d.noddraw=true` when running JOGL applications
on Windows; this system property disables the use of DirectDraw by
Java2D. There are driver-level incompatibilities between DirectDraw
and OpenGL which manifest themselves as application crashes, poor
performance, bad flickering, and other artifacts. This poor behavior
may exhibit itself when OpenGL and DirectDraw are simply used in the
same application, not even just in the same window, so disabling
Java2D's DirectDraw pipeline and forcing it to use its GDI pipeline is
the only way to work around these issues. Java Web Start applications
may set this system property by adding the following line to the
`<resources>` section of the JNLP file:

```
<property name="sun.java2d.noddraw" value="true"/>
```

There is a serious memory leak in ATI's OpenGL drivers which is
exhibited on Windows XP on Mobility Radeon 9700 hardware. It's
possible it will be present on other hardware as well though it was
not reproducible at the time of this writing on desktop Radeon
hardware or older ATI mobile chips. The bug is documented in JOGL Issue
166 and a bug has been filed with ATI. You can confirm the
presence of the bug either with the test case in that bug report or by
simply running the Gears demo; if the process size grows over time in
the Task Manager, the memory leak is present on your hardware. For the
time being, you can work around this memory leak by specifying the
system property `-Djogl.GLContext.nofree` on the command
line when launching your JOGL applications. There is no good
general-purpose workaround for this bug which behaves well on all
hardware.

### Linux

The Sun JDK "compatibility" RPMs (java-1.5.0-sun-compat,
java-1.6.0-sun-compat) provided by jpackage.org are incompatible with
JOGL. These RPMs symlink an internal JDK directory to /usr/lib, which
overrides how both NVidia and ATI currently provide their drivers to
some Linux distributions, which is through an override in
/etc/ld.so.conf (actually, in /etc/ld.so.conf.d). The implicit
presence of /usr/lib on LD\_LIBRARY\_PATH forces the /usr/lib/libGL.so.1
version of OpenGL to be used, which is typically Mesa and which will
provide only software rendering.

Unfortunately the JPackage maintainers have so far been unreceptive to
changing their installation mechanism; see this
mailing list posting. Until this is resolved, we strongly
discourage the use of the JPackage installers for the Sun JDK.
Instead, download the JRE or JDK installers directly from Sun's
website.

Archived forum postings illustrating this problem are here
and here.

### Solaris, Linux (X11 platforms)

Support has been added to the JOGL library for allowing multiple
threads to each have an OpenGL context current simultaneously, for
example to implement multi-head CAVE-like environments. Normally a
global AWT lock is held between calls to GLContext.makeCurrent() /
release() for on-screen heavyweight contexts (for example, those
associated with a Canvas or GLCanvas). We have found this to be
necessary for stability purposes on all supported X11 platforms, even
with relatively robust drivers such as those from NVidia.

To enable multiple GLContexts to be made current simultaneously on X11
platforms, specify the command line argument
`-Djogl.GLContext.optimize` when starting the JVM. Note
that this may incur robustness problems, in particular when resizing
or moving windows. We have also found that ATI's proprietary drivers
do not work at all with this flag, apparently because they cause GLX
tokens to be sent to the X server for various GL calls even for direct
contexts. For this reason if the GLX vendor is ATI then this flag
currently has no effect.

### Mac OS X

There are some problems with visual artifacts and stability problems
with some of the Jogl demos on Mac OS X. It appears that at least some
of these problems are due to bugs in Apple's OpenGL support. Bugs have
been filed about these problems and it is hoped they will be addressed
in the near future.

The Mac OS X port of Jogl, in particular the GL interface and its
implementation, can be used either with the provided GLCanvas widget
or with the Cocoa NSOpenGLView. In order to use it with Cocoa the
following steps should be taken:

- Create an "external" OpenGL context using the
  `GLDrawableFactory.createExternalGLContext()` API. The
  context object must be created while a real underlying OpenGL context
  is current.- Fetch the GL instance out of the context using getGL() as usual.
    Only use the GL instance when the OpenGL context from the NSOpenGLView
    is current.

**NOTE:** the Cocoa interoperability has not been retested
recently, though similar interoperability has been tested on other
platforms. Please report any problems found with using Jogl with an
NSOpenGLView.

The following issues remain with the Mac OS X port:

- Due to the mechanism by which the Cocoa graphics system selects
  OpenGL pixel formats, the GLCapabilitiesChooser mechanism can not be
  implemented on Mac OS X as on other platforms. Currently the
  underlying Cocoa pixel format selection is used on an
  NSOpenGLPixelFormat derived from the settings in the GLCapabilities,
  and the GLCapabilitiesChooser is ignored.

## Version History

JOGL's version history can be found online in the "JOGL Release
Information" thread in the JOGL forum. Comments about the 1.1
release train are in the thread "JOGL 1.1
released".
